# Applications, phytochemistry, pharmacological effects, pharmacokinetics, toxicity of *Scutellaria baicalensis* Georgi. and its probably potential therapeutic effects on COVID-19: a review

**DOI:** 10.1186/s13020-020-00384-0

**Published:** 2020-09-25

**Authors:** Jia-Wen Song, Jia-Ying Long, Long Xie, Lin-Lin Zhang, Qing-Xuan Xie, Hui-Juan Chen, Mao Deng, Xiao-Fang Li

**Affiliations:** School of Pharmacy, Chengdu University of Traditional Chinese Medicine, No. 1166, Liutai Avenue, Chengdu, 611137 China

**Keywords:** SB applications, Phytochemistry, Pharmacological effects, Pharmacokinetics, Toxicity, Treating COVID-19

## Abstract

*Scutellaria baicalensis* Georgi. (SB) is a common heat-clearing medicine in traditional Chinese medicine (TCM). It has been used for thousands of years in China and its neighboring countries. Clinically, it is mostly used to treat diseases such as cold and cough. SB has different harvesting periods and processed products for different clinical symptoms. Botanical researches proved that SB included in the Chinese Pharmacopoeia (1st, 2020) was consistent with the medicinal SB described in ancient books. Modern phytochemical analysis had found that SB contains hundreds of active ingredients, of which flavonoids are its major components. These chemical components are the material basis for SB to exert pharmacological effects. Pharmacological studies had shown that SB has a wide range of pharmacological activities such as antiinflammatory, antibacterial, antiviral, anticancer, liver protection, etc. The active ingredients of SB were mostly distributed in liver and kidney, and couldn't be absorbed into brain via oral absorption. SB’s toxicity was mostly manifested in liver fibrosis and allergic reactions, mainly caused by baicalin. The non-medicinal application prospects of SB were broad, such as antibacterial plastics, UV-resistant silk, animal feed, etc. In response to the Coronavirus Disease In 2019 (COVID-19), based on the network pharmacology research, SB’s active ingredients may have potential therapeutic effects, such as baicalin and baicalein. Therefore, the exact therapeutic effects are still need to be determined in clinical trials. SB has been reviewed in the past 2 years, but the content of these articles were not comprehensive and accurate. In view of the above, we made a comprehensive overview of the research progress of SB, and expect to provide ideas for the follow-up study of SB.
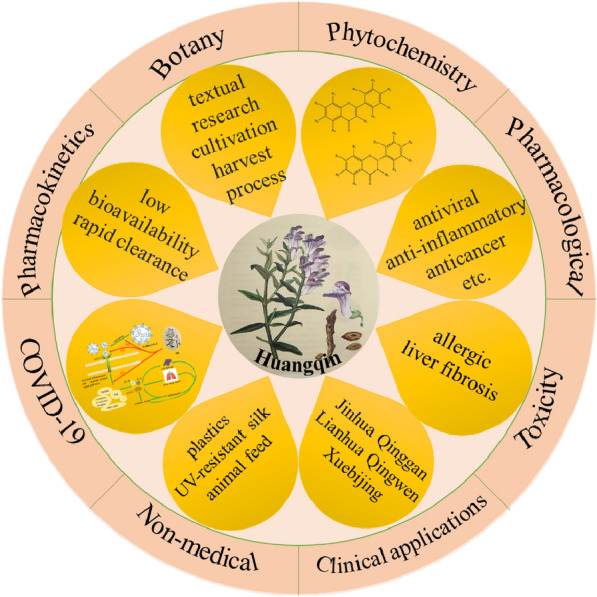

## Background

SB, a species in the genus *Scutellaria* (family *Lamiaceae*) which its dried root is a TCM recorded in ChP (1st, 2020) [1], often been called Huangqin or Skullcap. SB has been widely used for thousands of years by China and its neighbors. It mainly grows in temperate regions and tropical mountains (with an altitude of about 1300–3000 m), including China, Russia’s Eastern Siberia, Mongolia, North Korea, Japan, etc. [2]. The traditional therapeutic effects have been first recorded in the *Shennong Bencaojing (The Classic of Herbal Medicine)* [3], an existing original pharmacological monograph of China written by many medical scientists together during the Han dynasty. It narrated in detail that SB has “the effects of clearing away the pathology of heat and dampness, eliminating fire pathology, detoxifying, and preventing bleeding and fetal restlessness” [1].

SB contains various chemical components, such as various flavonoids, diterpenes, polyphenols, amino acids, volatile oils, sterols, benzoic acids, etc. [4, 5]. Flavonoids are the main components of SB. There are more than 110 kinds of flavonoids in its dried roots, including baicalin, baicalein, wogonoside, wogonin, etc. These are the main material basis for SB to play clinical effects. SB has antibacterial, antiviral, anti-inflammatory, anticancer, liver and nerve protective functions, etc. [6–8]. Because SB has a very wide range of pharmacological effects and clinical applications, more and more in-depth researches had been implemented. This paper is a comprehensive summary of the current researches on SB, including clinical and non-clinical applications, medicinal materials, botany, phytochemistry, pharmacology, pharmacokinetic characteristics, and toxicity. And then, we also discussed that SB has a certain potential effects in the treatment of the current major disease-COVID-19 based on the current network pharmacology researches. Its possible prevention and treatment mechanisms were discussed, but the exact therapeutic effects still need to be determined in the next step.

## Applications

### Clinical applications

SB is one of the 40 bulk medicinal materials and one of the famous “three yellow” in TCM [9]. It is a common medicine for treating cold, flu, fever, diarrhea, jaundice, headache, abdominal pain, drenching, etc. SB is frequently used in classical prescription, such as Mahuang Shengma decoction, Xiexin decoction and Huangqin decoction, ect [10]. In modern clinical applications, the quantity or dosage of TCM in prescriptions can be added or reduced to clearing away heat with different symptoms [11]. Compared with other antipyretic TCM, SB is more suitable for heat syndrome with fetal restlessness. There are about 477 prescriptions containing SB [12] for treating 153 main diseases, cough and cold are more frequently used. And after searching ChP. (4th, 2020) [13], for the treatment of cold, more than 30% of Chinese patent medicine contained SB active ingredients, and for clearing away heat and detoxifying more than 70% of them contained SB and its active ingredients. Common forms of these preparations include granule, pill, tablet, liquid preparation, capsule, etc. [14]. The same prescription may have different preparation forms, and the appropriate form should be selected according to the development of the disease.

### Non-clinical applications

A composite material contained SB had good biocompatibility and strong antibacterial activity [15]. Many components (e.g. baicalin, baicalein and wogonin) had reversible electronic shuttle activity for the extraction of bioenergy [16]. Microbial fuel cells contained baicalin and baicalein had higher ability of bioelectricity generation, and showed weaker biological toxicity during the process of generating electricity. Feed contained SB flavonoids improved the meat quality and antioxidant capacity of broilers and increased the activity of SOD, GSH and peroxidase in liver [17]. It is suggested that SB flavonoids can be added into agricultural feed to improve meat quality. A silk product contained baicalin was successfully prepared [18], and it had antibacterial, anti-oxidation and UV protection effects. In addition, it also showed an electrostatic effect. SBE had the effect of inhibiting the synthesis of melanin, and had good safety to the skin [19]. It can be used as a whitening ingredient in the beauty industry after more clinical trials based on human skin safety.

## Botanical researches

### Textual researches of botany

Genuine SB is the dried root of perennial herb SB of the *Labiaceae* family. It is widely distributed in Hebei, Shandong, Shanxi and other regions in China. It is suitable for growing in the dry sunny places such as the mountain top, hillside, forest edge and roadside with an altitude of 500–1500 m [20]. *Illustrated Pharmacopoeia* (*Ben Cao Tu Jing*) narrated that “The seedling is more than foot-long, the stem is as thick as chopsticks, and the leaves are clustered from all sides of the ground”. *Compendium of Materia Medica* (*Ben Cao Gang Mu*) also narrated that “Kuqin (rotten xylem) is an old root with hollow and yellow outside and black inside. Ziqin (strip types) is a new root with more compact inside. SB in southwest is hollow and black, contrast to SB in north is solid and deep yellow”. The former is mainly used to treat lung heat cough and the latter is mainly used to treat damp heat dysentery. However, there is no excessive differentiation in clinical use [21]. Therefore, the plant morphology of SB described by the ancients is consistent with that of SB used now [22].

### Harvesting and processing

In addition to the regulation of genetic factors and the influence of environmental conditions [23], the quality of effective components of TCM is also affected by harvesting and processing [24]. Studies have shown that there are differences in the content of SB in spring and autumn [25]. The best harvest time of SB is determined by the highest content of baicalin, wogonoside, baicalein and scutellarin, most of which are concentrated in September [26]. According to the growth condition of SB and the dynamic accumulation of baicalin, there was a significant increase of baicalin in triennial compared with biennials in the root [20]. Therefore, the harvest time of SB is mainly concentrated in autumn, and the quality of triennial one is the best.

The traditional processing of SB needs to bump away the rough skin. However, the key enzymes for the synthesis of flavonoids were found to be mainly concentrated in the phloem of the root according to spatial imaging [27]. The content of baicalin in cortex is the highest, which affects the quality of SB. Therefore, it is suggested that the above-mentioned step should be cancelled in the modern processing of SB, so as to ensure the quality of SB [28, 29]. The total amount of baicalin and flavonoids in the process of drying shows a change trend of inverted “V”, which may be related to the physiological mechanism of anti-drought stress of SB root itself [26, 30]. This showed that the drying time should not be too long, otherwise the content of active ingredients will be reduced. At present, there are different processed products of SB, such as wine, fried and charcoal SB respectively. Different processed products of SB play different roles in the prescription [31]. The commonly used processed products of SB are raw materials, which can play the role of clearing away heat and detoxification. Wine SB can play the role of tonifying and moistening in addition to clearing away heat.

### Cultivation conditions

By controlling the cultivation conditions, the content of the effective components of planted SB can be improved. Yuan et al. found that the lack of water can cause the hormone metabolism in SB and affect the synthesis of baicalin and other flavonoids [32]. The synthesis of different flavonoids in SB will change differently under UV-B irradiation [33]. For example, the content of chrysin, scutellarin, baicalin and tectoridin would changed under different intensity UV-B irradiation. Free salicylic acid has a great effect on the accumulation of baicalin in the growth of SB, but it has little effect on baicalein [34]. Blue, white and red light has different effects on the content of flavonoids [35]. The content of baicalin increased with the extension of illumination time, and its content reached the maximum under blue light. These studies indicated that the content of water, free salicylic acid, UV-B irradiation, and different light time and intensity could be regulated to change the content of flavonoids in the cultivation process of SB.

## Phytochemistry

**Fig. 1:**
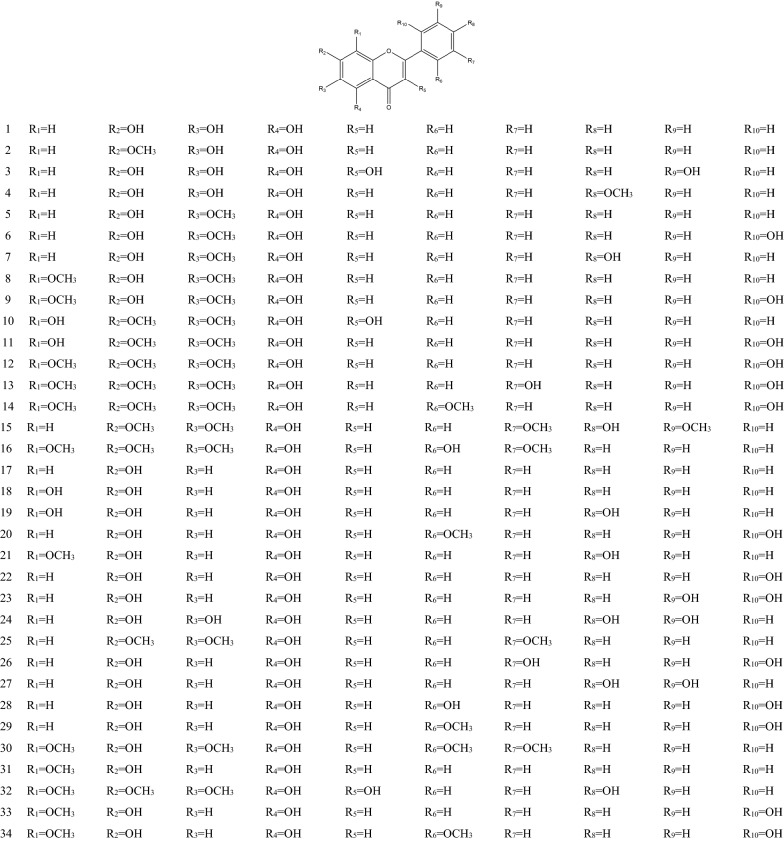

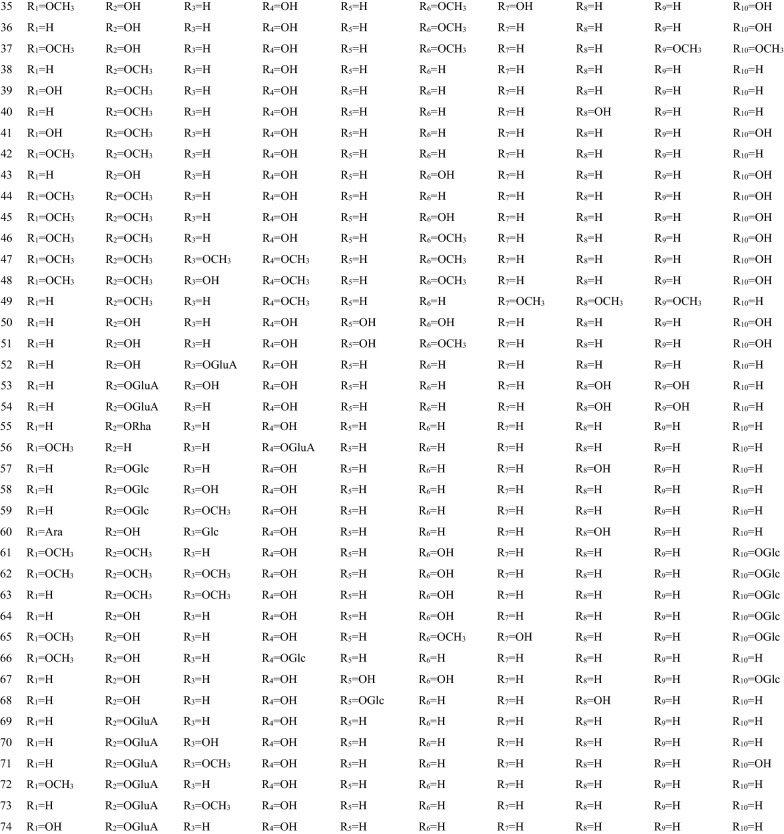

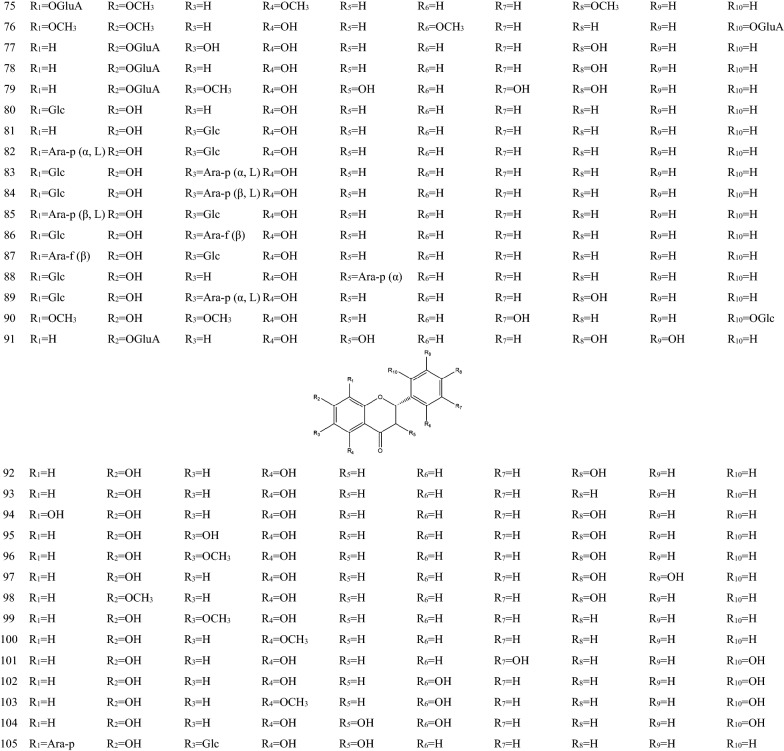

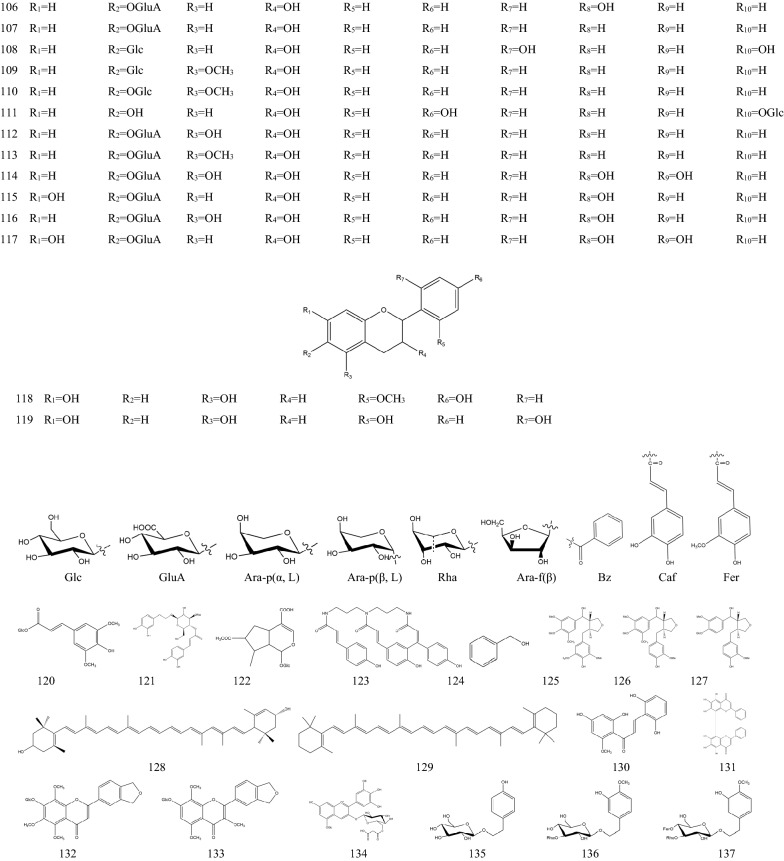

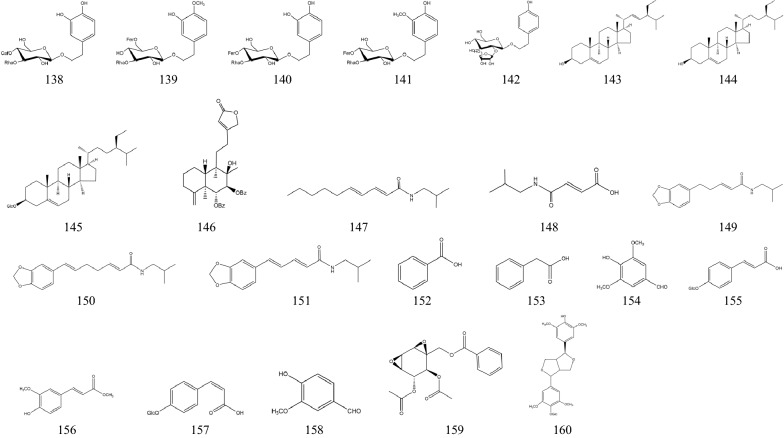
Chemical structure of components of SB

**Fig. 2:**
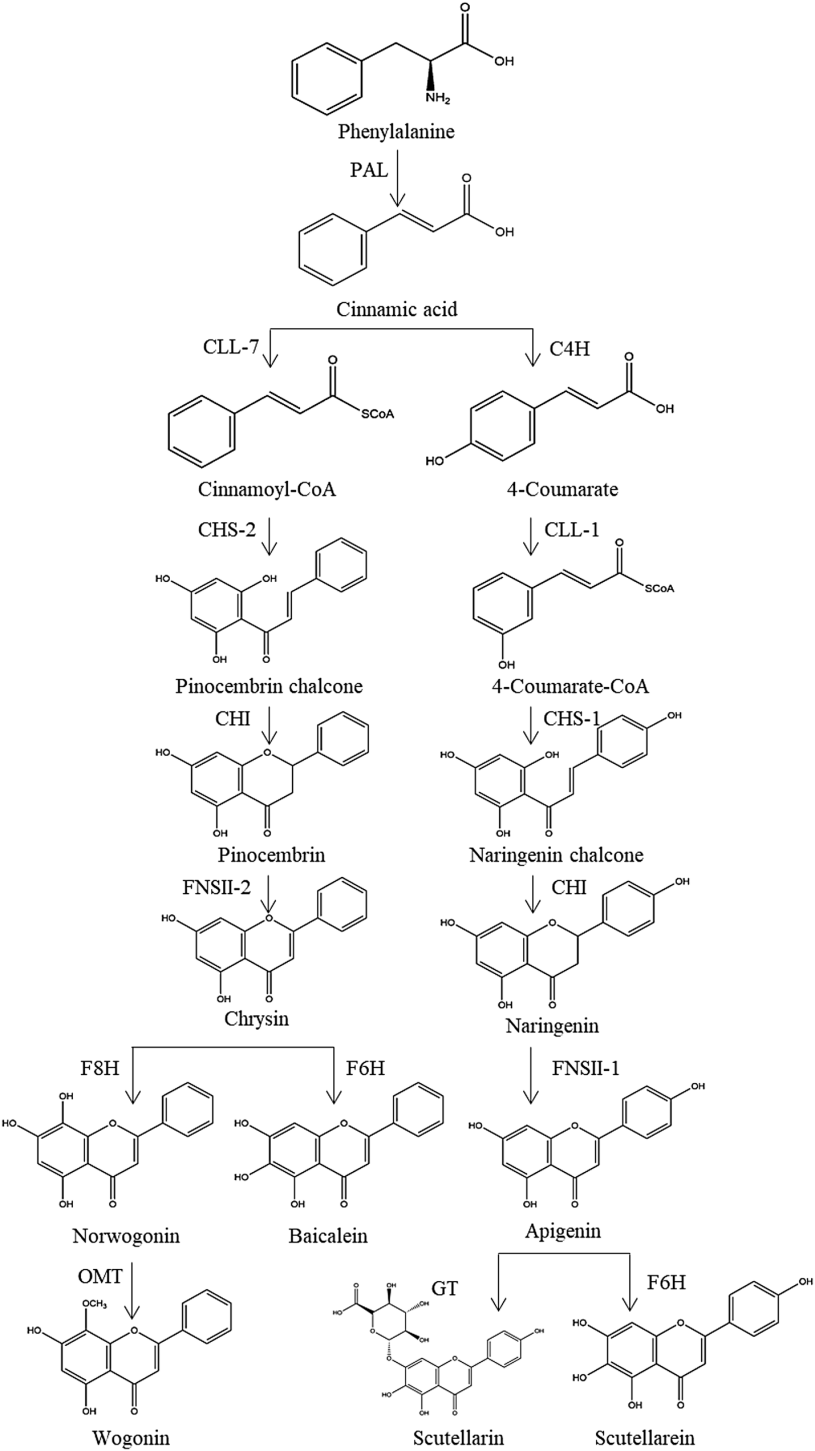
Main synthetic routes of flavonoids of SB

In 1970s, many chemical components of SB had been separated one after another. These components are the material basis for SB to play a wide range of pharmacological actions [36]. There are hundreds of ingredients in SB (shown in Table 1 and Fig. 1), in addition to flavonoids, it also contains volatile oils, terpenoids, polysaccharides, phenylethyl, amino acids, sterols, starch, alkaloids, organic acids and trace elements [40]. Flavonoids are the major components of SB. There are more than 100 kinds of flavonoids in SB, it is important to note that pharmacological activities of SB take place due to the presence of specific 4′-deoxyflavones such as chrysin, baicalein, wogonin and their glycosides (baicalin and wogonoside) [41]. The key enzymes for the synthesis of these compounds are mainly found in roots, including PAL, C4H, CLL, CHS, CHI, FNSII, MT, GT, FH and OMT, different flavonoid synthetases are encoded by different genes [6, 42, 43]. The main synthetic routes of flavonoids were shown in Fig. 2.

**Table 1:** The components of SB

No.	Name	Molecular formula	Weight	Plant part	Ref.
1	Baicalein (5,6,7-Trihydroxyflavone)	C_15_H_10_O_5_	270	Root Hairy Root	[37]
2	Methoxybaicalein (5,6-Dihydroxy-7-methoxyflavone)	C_16_H_12_O_5_	284	Root	[117]
3	Scutellarein (5,6,7,4′-Tetrahydroxyflavone)	C_15_H_10_O_6_	286	Root	[39]
4	5,6,7-Trihydroxy-4′-methoxyflavone	C_16_H_12_O_6_	300	Aerial part	[38]
5	Oroxylin A (5,7-Dihydroxy-6-methoxyflavone)	C_16_H_12_O_5_	284	Root	[37]
6	Tenaxin II (5,7,2′-Trihydroxy-6-methoxyflavone)	C_16_H_12_O_6_	300	Root	[38]
7	5,7,4′-Trihydroxy-6-methoxyflavone	C_16_H_12_O_6_	300	Aerial part	[38]
8	5,7-Dihydroxy-6,8-dimethoxyflavone	C_17_H_14_O_6_	314	Root	[38]
9	5,7,2′-Trihydroxy-6,8-dimethoxyflavone	C_17_H_14_O_7_	330	Root	[38]
10	5,8-Dihydroxy-6,7-dimethoxyflavone	C_17_H_14_O_6_	314	Root	[38]
11	5,8,2′-Trihydroxy-6,7-dimethoxyflavone	C_17_H_14_O_7_	330	Root	[38]
12	Tenaxin I (5,2′-Dihydroxy-6,7,8-trimethoxyflavdne)	C_18_H_16_O_7_	344	Root	[38]
13	5,2′,5′-Trihydroxy-6,7,8-trimethoxyflavone	C_18_H_16_O_8_	360	Root	[38]
14	Skullcapflavone II (5,6′-Dihydroxy-6,7,8,2′-tetramethoxyflavone)	C_19_H_18_O_8_	374	Root Hairy Root	[37]
15	5,4′-Dihydroxy-6,7,3′,5′-tetramethoxyflavone	C_19_H_18_O_8_	374	Aerial part	[38]
16	5,2′-Dihydroxy-6,7,8,3′-tetramethoxyflavone	C_19_H_18_O_8_	374	Hairy Root	[38]
17	Chrysin (5,7-Dihydroxyflavone)	C_15_H_10_O_4_	254	Root Aerial part	[37]
18	Norwogonin (5,7,8-Trihydroxyflavone)	C_15_H_10_O_5_	270	Root	[38]
19	lsoscutellarein (5,7,8,4′-Tetrahydroxyflavone)	C_15_H_10_O_6_	286	Aerial part	[38]
20	Apigenin (5,7,4′-Trihydroxyflavone)	C_15_H_10_O_5_	270	Root Aerial part	[39]
21	4′-Hydroxywogonin (5,7 4′-Trihydroxy-8-methoxyflavone)	C_16_H_12_O_6_	300	Root	[38]
22	2′-Hydroxychrysin (5,7,2′-Trihydroxyflavone)	C_15_H_10_O_5_	270	Root	[38]
23	5,7,2′,3′-Tetrahydroxyflavone	C_15_H_10_O_6_	286	Root	[38]
24	6-Hydroxyluteolin	C_15_H_10_O_7_	292	Whole plant	*[*39*]*
25	Salvigenin	C_18_H_16_O_7_	344	Root	[38]
27	Luteolin	C_15_H_10_O_6_	286	Whole plant	[39]
26	5,7,2′,5′-Tetrahydroxyflavone	C_15_H_10_O_6_	286	Root	[38]
28	5,7,2′,6′-Tetrahydroxyflavone	C_15_H_10_O_6_	286	Root	[38]
29	5,7,6′-Trihydroxy-2′-methoxyflavone	C_16_H_12_O_6_	300	Root	[38]
30	5,7-Dihydroxy-6,8,2′,3′-tertramethoxyflavone	C_19_H_18_O_7_	358	Root	[38]
31	Wogonin (5,7-Dihydroxy-8-methoxyflavone)	C_16_H_12_O_5_	284	Root Aerial part Hairy Root	[37]
32	3,5,4′-Trihydroxy-6,7,8-trimethoxyflavone	C_18_H_16_O_8_	360	Whole plant	[39]
33	Scutevulin (5,7,2′-Trihydroxy-8-methoxyflavone)	C_16_H_12_O_6_	300	Root	[38]
34	5,7,6′-Trihydroxy-8,2′-dimethoxyflavone	C_17_H_14_O_7_	330	Root	[37]
35	Viscidulin III (5,7,3′,6′-Tetrahydroxy-8,2′-dimethoxyflavone)	C_17_H_14_O_8_	346	Root	[38]
36	5,7,2′-Trihydroxy-6′-methoxyflavone	C_16_H_12_O_6_	300	Root	[38]
37	5,7-Dihydroxy-8,2′,3′,6′-tetramethoxyflavone	C_19_H_18_O_8_	374	Root	[38]
38	7-Methoxychrysin (5-Hydroxy-7-methoxyflavone)	C_16_H_12_O_4_	268	Aerial part	[38]
39	5,8-Dihydroxy-7-methoxyflavone	C_16_H_12_O_5_	284	Root	[38]
40	Genkwanin (5.,4′-Dihydroxy-7-methoxyflavone)	C_16_H_12_O_5_	284	Aerial part	[38]
41	5,8,2′-Trihydroxy-7-methoxyflavone	C_16_H_12_O_6_	300	Root	[38]
42	7-O-Methylwogonin (5-Hydroxy-7,8-dimethoxyflavone)	C_17_H_14_O_5_	298	Root	[38]
43	5,7,4′-Trihydroxy-8-methoxyflavone	C_17_H_12_O_6_	312	Root	[38]
44	Skullcapflavone I (5,2′-Dihydroxy-7,8-dimethoxyflavone)	C_17_H_14_O_6_	314	Root Hairy Root	[38]
45	Viscidulin II (5,2′,6′-Trihydroxy-7,8-dimethoxyflavone)	C_17_H_14_O_7_	330	Root	[38]
46	Rivularin (5,6′-Dihydroxy-7,8,2′-trimethoxyflavone)	C_18_H_16_O_7_	344	Root Hairy Root	[38]
47	6′-Hydroxy-5,6,7,8,2′-pentamethoxyflavone	C_20_H_20_O_8_	388	Root	[38]
48	6,б′-Dihydroxy-5,7,8,2′-tetramethoxyflavone	C_19_H_18_O_8_	374	Root	[38]
49	5,7,3′4′,5′-Pentamethoxyflavone	C_20_H_20_O_7_	372	Aerial part	[38]
50	Viscidulin I (5,7,2′,6′-Tetrahydroxyflavonol)	C_15_H_10_O_7_	302	Root	[37]
51	5,7,6′-Trihydroxy-2′-methoxyflavonol	C_16_H_12_O_7_	316	Root	[38]
52	Baicalein 6-O-glucuronide	C_21_H_18_O_11_	446	Whole plant	[39]
53	6-Hydroxyluteolin 7-O-glucoronide	C_21_H_18_O_13_	478	Whole plant	[39]
54	Luteolin 7-O-glucuronide	C_21_H_18_O_12_	462	Whole plant	[39]
55	Apigenin 7,4′-di-O-rhamnoside	C_21_H_20_O_8_	400	Whole plant	[39]
56	8-Methoxy-5-O-glucosideflavone	C_22_H_20_O_10_	444	Root	[38]
57	Apigenin 7-O-β-D-glucoside	C_21_H_20_O_10_	432	Aerial part	[39]
58	Baicalein 7-O-β-D-glucoside	C_21_H_20_O_10_	432	Root Aerial part	[37]
59	Oroxylin A 7-O-β-D-glucoside	C_21_H_20_O_10_0	446	Aerial part Root	[37]
60	Apigenin 6-C-glucosyl-8-C-arabinoside	C_26_H_28_O_14_	564	Whole plant	[39]
61	5,6′-Dihydroxy-7,8-dimethoxyflavone 2′-O-β-D-glucoside	C_23_H_24_O_12_	492	Root Hairy Root	[37]
62	5,6′-Dihydroxy-6,7,8-trimethoxyflavone 2′-O-β-D-glucoside	C_24_H_26_O_13_	522	Root	[38]
63	5,6′-Dihydroxy-6,7-dimethoxyflavone 2′-O-β-D-glucoside	C_23_H_24_O_12_	492	Root Hairy Root	[38] [38]
64	5,7,6′-Trihydroxyflavone 2′-O-β-D-glucoside	C_21_H_20_O_11_	448	Hairy Root	[37]
65	Viscidulin III 6′-O-β-D-glucoside	C_23_H_24_O_13_	508	Root Hairy Root	[37]
66	Wogonin 5-O-β-D-glucoside	C_22_H_22_O_10_	446	Root	[37]
67	3,5,7,6′-Tetrahydroxyflavone 2′-O-β-D-glucoside	C_21_H_20_O_12_	464	Root	[38]
68	Kaempferol 3-O-β-D-glucoside	C_21_H_20_O_11_	448	Aerial part	[38]
69	Chrysin 7-O-β-d-glucuronide	C_21_H_18_O_10_	430	Root Aerial part	[37]
70	Baicalin (5,6-Dihydroxyflavone 7-O-β-D-glucuronide)	C_21_H_18_O_11_	446	Root Aerial part Hairy Root	[37]
71	5,2′-Dihydroxy-6-methoxyflavone 7-O-β-D-glucuronide	C_22_H_20_O_12_	476	Root	[37]
72	Wogonoside (Wogonin 7-O-β-D-glucuronide)	C_22_H_20_O_11_	460	Root Hairy Root	[37]
73	Oroxyloside (Oroxylin A 7-O-β-D-glucuronide)	C_22_H_20_O_11_	460	Root	[37]
74	Norwogonin 7-O-β-D-glucuronide (5,8-dihydroxyflavone 7-O-β-D-glucuronide)	C_21_H_18_O_11_	446	Root	[37]
75	Isoscutellarein 8-O-β-D-glucuronide	C_24_H_24_O_12_	504	Leaf	[38]
76	5-Hydroxy-7,8,6′-trimethoxyflavone 2′-O-β-D-glucuronide	C_24_H_24_O_13_	520	Hairy Root	[38]
77	Scutellarin	C_21_H_18_O_12_	462	Root	[37]
78	Apigenin 7-O-β-D-glucuronide	C_21_H_18_O_11_	446	Aerial part	[39]
79	Patuletin 7-O-β-D-glucuronide (3,5,3′,4′-Tetrahydroxy-6-methoxyflavone 7-O-β-D-glucuronide)	C_22_H_20_O_14_	508	Root	[38]
80	Chrysin 8-C-β-D-glucoside	C_21_H_20_O_9_	416	Root	[37]
81	Chrysin 6-C-β-d-glucoside	C_21_H_20_O_9_	416	Root	[38]
82	Chrysin 6-C-β-D-glucoside-8-C-α-l-arabinopyranoside	C_26_H_28_O_13_	548	Root Hairy Root	[37]
83	Chrysin 6-C-α-l-arabinopyranoside-8-C-β-D-glucoside	C_26_H_2_8O_13_	548	Root Hairy Root	[37]
84	Chrysin 6-C-β-l-arabinopyranoside-8-C-β-D-glucoside	C_26_H_28_O_13_	548	Root	[39]
85	Chrysin 6-C-β-D-glucoside-8-C-β-l-arabinopyranoside	C_26_H_28_O_13_	548	Root	[39]
86	Chrysin 6-C-β-arabinofuranoside-8-C-β-D-glucoside	C_26_H_28_O_13_	548	Root	[39]
87	Chrysin 6-C-β-d-glucoside-8-C-β-arabinofuranoside	C_26_H_28_O_13_	548	Root	[39]
88	Chrysin 3-C-α-arabinopyranoside-8-C-β-D-glucoside	C_26_H_28_O_13_	548	Root	[39]
89	Apigenin 6-C-α-l-arabinopyranoside-8-C-β-D-glucoside(isoschaftoside)	C_26_H_28_O_14_	564	Aerial part	[39]
90	Viscidulin III-2′-O-β-D-glucopyranoside	C_23_H_22_O_14_	522	Root	[38]
91	Quercetin 3-glucuronide	C_21_H_18_O_13_	478	Whole plant	[39]
92	Naringenin	C_15_H_12_O_5_	272	Whole plant	*[*39*]*
93	Pinocembrin	C_15_H_12_O_4_	256	Whole plant	[39]
94	lsocarthamidin ((2S)-5,7,8,4′-Tetrahydroxyflavanone)	C_15_H_12_O_6_	288	Leaf Root	[39]
95	Carthamidin (2S)-5,6,7,4′-Tetrahydroxyflavanone)	C_15_H_12_O_6_	288	Leaf Root	[39]
96	(2S)-5,7,4′-Trihydroxy-6-methoxyflavanone	C_16_H_14_O_6_	302	Root	[38]
97	(+)-Eriodictyol ((2S)-5,7,3′,4′-Tetrahydroxyflavanone)	C_15_H_12_O_6_	288	Root	[38]
98	(2S)-5,4′-Dihydroxy-7-methoxyflavanone	C_16_H_14_O_5_	286	Aerial part	[38]
99	Dihydrooroxylin A ((2S)-5,7-Dihydroxy-6-methoxyflavanone)	C_16_H_14_O_5_	286	Root	[38]
100	(2S)-7-Hydroxy-5-methoxyflavanone	C_16_H_14_O_4_	270	Root	[38]
101	(2S)-5,7,2′,5′-Tetrahydroxyflavanone	C_15_H_12_O_6_	288	Root	[38]
102	(2S)-5,7,2′,6′-Tetrahydroxyflavanone	C_15_H_12_O_6_	288	Root	[37]
103	(2S)-7,2′,6′-Trihydroxy-5-methoxyflavanone	C_16_H_14_O_6_	302	Root	[38]
104	(2R,3R)-3,5,7,2′,6′-Pentahydroxyflavanone	C_15_H_12_O_7_	304	Root	[37]
106	Naringenin 7-O-glucoronide	C_21_H_20_O_11_	448	Whole plant	[39]
107	Pinocembrin 7-O-glucoronide	C_21_H_20_O_10_	432	Whole plant	[39]
108	(2S) -5,7,2′,5′-Tetrahydroxyflavanone-7-O-β-D-glucopyranoside	C_21_H_22_O_10_	434	Root	[38]
109	(2S)-5,7-Dihydroxy-6-methoxyflavanone-7-O-β-D-glucopyranoside	C_22_H_22_O_10_	446	Root	[38]
110	(2S)-5-Hydroxy-6-methoxyflavanone 7-O-β-D-glucoside	C_22_H_24_O_10_	448	Root	[38]
111	(2S)-5,7,6′-Trihydroxyflavanone 2′-O-β-D-glucoside	C_21_H_22_O_11_	450	Root	[37]
112	Dihydrobaicalin ((2S)-5,6-Dihydroxyflavanone 7-O-β-D-glucuronide)	C_21_H_20_O_11_	448	Root	[38]
113	(2S)-5-Hydroxy-6-methoxyflavanone 7-O-β-D-glucuronide	C_22_H_22_O_11_	462	Root	[37]
114	(2S)-5,6,3′,4′-Tetrahydroxyflavanone 7-O-β-D-glucuronide	C_21_H_20_O_13_	480	Aerial part	[38]
115	Isocarthamidin-7-O-β-D-glucuronide ((2S)-5,8,4′-Trihydroxyflavanone 7-O-β-D-glucuronide)	C_21_H_20_O_12_	464	Aerial part	[39]
116	Carthamidin 7-O-β-D-glucuronide (Dihydroscutellarein 7-O-β-D-glucuronide, Scutellarin B)	C_21_H_20_O_12_	464	Aerial part	[39]
117	(2S)-5,8,3′,4′-Tetrahydroxyflavanone 7-O-β-D-glucuronide	C_21_H_20_O_13_	480	Aerial part	[38]
118	4′,5,7-Trihydroxy-6-methoxyflavanone	C_16_H_15_O_5_	287	Root	[38]
119	2′,6′,5,7-Tetrahydroxyflavanone	C_15_H_13_O_5_	273	Root	[38]
120	Sinapoyl hexoside	C_27_H_22_O_10_	386	Whole plan	*[*39*]*
121	Verbascoside	C_29_H_36_O_15_	624	Whole plant	[39]
122	7-O-Acetylloganic acid	C_28_H_26_O_10_	402	Whole plant	*[*39*]*
123	N^1^,N^5^,N^10^-Tri-p-(E,E,E)-coumaroylspermidine	C_33_H_33_O_6_N_3_	567	Whole plant	[39]
124	Benzyl alcohol	C_7_H_8_O	108	Root	[36]
125	4′-(β-D-glucopyranosyloxy)-3,3′,5,5′-tetramethoxy-9,9′-epoxylignane-4, 7′-diol	C_30_H_45_O_13_	613	Root	[36]
126	4′-(β-D-glucopyranosyloxy)-3,3′,5′-trimethoxy-9,9′-epoxylignane-4, 7′-diol	C_29_H_43_O_12_	583	Root	[36]
127	4′-(β-D-glucopyranosyloxy)-3,3-dimethoxy-9,9′-epoxylignane-4,7′-diol	C_28_H_41_O_11_	553	Root	[36]
128	Lutein	C_40_H_55_O_2_	567	Root	[36]
129	β-Carotene	C_40_H_56_	536	Root	[36]
130	2,6,2′,4′-Tetrahydroxy-6′-methoxychalcone	C_16_H_14_O_6_	302	Root	[38]
131	8,8″-Bibaicalein	C_30_H_18_O_10_	538	Root	[38]
132	5,6,8-Trimethoxy-3′,4′-methylenedioxyflavone 7-O-β-D-glucoside	C_26_H_28_O_12_	532	Root	[38]
133	3,5,8-Trimethoxy-3′,4′-methylenedioxyflavone 7-O-β-D-glucoside	C_26_H_28_O_12_	532	Root	[38]
134	Delphinidin 3-O-(6-O-malonyl)-β-d-glucoside-5-O-β-D-glucoside	C_30_H_33_O_20_	713	Flower	[38]
135	Salidroside (4-Hydroxy-β-phenylethyl-β-D-glucoside)	C_14_H_20_O_7_	300	Hairy Root	[38]
136	Darendoside B	C_21_H_32_O_12_	476	Root	[38]
137	Martynoside (2-(3-Hydroxy-4-methoxyphenyl) ethyl-1-O-α-l-rhamnosyl(1 → 3)-β-D-(4-feruloyl)-glucoside)	C_31_H_40_O_15_	652	Hairy Root Root	[38]
138	Acteoside	C_29_H_36_O_15_	624	Hairy Root Root	[37]
139	Isomartynoside	C_31_H_40_O_15_	652	Root	[38]
140	Leucosceptoside A	C_30_H_38_O_15_	638	Hairy Root Root	[38]
141	Cistanoside D	C_31_H_40_O_15_	652	Root	[37]
142	Darendoside A	C_19_H_28_O_11_	432	Root	[38]
143	Stigmasterol	C_29_H_48_O	412	Root	[38]
144	β-Sitosterol	C_29_H_50_O	414	Root	[36]
145	Daucosterin	C_35_H_60_O_6_	576	Root	[38]
146	Scutebaicalin (6α,7β-dibenzoyloxy-8β-hydroxy-neo-cleroda-4(18),13-dien-15,16-olide)	C_34_H_38_O_7_	558	Aerial part	[38]
147	Pellitorine	C_14_H_25_NO	223	Root	[38]
148	(E)-4-[(2-methylpropyl) amino]-4-oxo-2-butenoic acid	C_8_H_13_NO_3_	171	Root	[38]
149	Dihydropiperlonguminine	C_16_H_21_NO_3_	275	Root	[38]
150	Futoamide	C_18_H_23_NO_3_	301	Root	[38]
151	Piperlonguminine	C_16_H_19_NO_3_	273	Root	[38]
152	Benzoic acid	C_7_H_6_O_2_	122	Root	[38]
153	Phenyl acetic acid	C_8_H_8_O_2_	136	Root	[38]
154	Syringaldehyde	C_9_H_10_O_4_	182	Root	[38]
155	4-O-β-D-glucosyl-trans-p-coumaric acid	C_15_H_18_O_8_	326	Root	[38]
156	Ferulic acid methyl ester	C_11_H_12_O_4_	208	Root	[38]
157	4-O-β-D-glucosyl-cis-p-coumaric acid	C_15_H_18_O_8_	326	Root	[38]
158	Vanillin	C_8_H_8_O_3_	152	Root	[38]
159	(+)-Crotepoxide	C_18_H_18_O_8_	362	Root	[38]
160	(+)-Syringaresinol-O-β-D-glucoside	C_28_H_36_O_13_	580	Root	[38]

Volatile oils, as secondary metabolites of plants, are widely distributed in the whole plant. Acetophenone, 1-phenyl-1,3-butanediol, palmitic acid and oleic acid are the most abundant [44]. The content and composition of volatile oils in different parts are different [45]. For example, germacrene D, bornyl acetate, diphenylamine and hexadecanoic acid are the primary oils in flowers, stem leaves, roots and seeds respectively. Terpenoids are mainly distributed in the aboveground part and not exist in seeds [46]. They have antibacterial, antiviral, antipyretic, analgesic and anti-inflammatory effects [47].

The polysaccharides of SB are water-soluble, mainly composed of arabinose, galactose, glucose and some differential branched dextran [48–50]. At present, a group of new polysaccharides SP1-1 with molecular weight of 4.56 × 10^5^ Da was identified and had anti-inflammatory effect [51], which is mainly composed of mannose, ribose, glucuronic acid, glucose, xylose and arabinose (the molar ratio were 2.14:3.61:1:2.86:5.98:36.39).

There are 14 kinds of amino acids in SB, of which proline is the highest, accounting for 80% [52]. Sterols contain α-spinasterol and β-sitosterol [40], among them, β-sitosterol has been proved to be effective for anorexia. Phenolic compounds, as organic acid components of medicinal plants, such as citric acid, tartaric acid, malonic acid, etc., have influence on the growth of plants [53]. Exception for lutein and β-carotene [54], there also are phytoene, zeaxanthin and ξ-carotene. And there are 3 lignin glycosides in SB [55]. Platinum is is mainly concentrated in roots, which its content related to the growth of SB [56]. Recently, a new actinomycete isolated from the cortex of SB [57], named *Brachybacterium endophyticum* sp. nov., was identified as a gram-positive, aerobic, coccus-shaped, non-spore-forming actinobacterium. Whether the existence and growth of the bacteria will affect the synthesis of active ingredients, it maybe as a new research point of SB.

## Pharmacological effects (Shown in Table 2)

### Anti-inflammatory

According to clinical applications, SB is widely used in anti-inflammatory effect. Many diseases can lead to inflammation. In the process of inflammation, all kinds of inflammatory mediators will be produced, such as iNOS, COX-2, IL-6, TNF-α, etc. These mediators can aggravate the inflammatory and then further expand into a vicious cycle [58]. It has been confirmed that the flavonoids of SB can inhibit the release of many inflammatory factors [59]. A new polysaccharide was extracted from SB, and it also could significantly inhibit the level of pro-inflammatory cytokines in serum, including TNF-α, IL-1 β, IL-18, etc. [51]. For inflammatory cells, baicalein can induce their apoptosis, thus reducing the release of inflammatory factors and the invasion of normal cells under the infiltration of inflammatory cells [60]. Inflammation is associated with the expression of many proteins, such as COX-2, vimentin, annexin A1, annexin A2, etc. And flavonoids in SB can inhibit the expression of these proteins to achieve anti-inflammatory effect [61]. The signaling pathways of PPARγ and NF-κB were also associated with the occurrence of inflammatory. SBE could inhibit LPS induced AET II cell inflammatory response by inhibiting NF-κB, MAPK and phosphatidylinositol-3 kinase signaling pathways [62], and increased the cell viability. To sum up, SB play an anti-inflammatory role mainly by inhibiting the release of inflammatory factors and the expression of inflammatory related proteins.

### Antibacterial and antimicrobial

There were studies showed that SB also has antibacterial and anti microbial effects. The SBWE could significantly inhibit the reproduction of *Toxoplasma gondii* in 24, 48 and 72 h [63]. Norwogonin has strong inhibitory effect on multidrug resistant *Acinetobacter baumannii* [64]. SB can significantly inhibit the up-regulation of IL-1β and IL-8 level caused by *Propionibacterium acnes* via inactivation of the MAPK and NF-κB signaling pathways. The main effective components are wogonin and wogonoside [65]. Baicalin had a dose-dependent inhibitory effect on the expression of LasA protease, LasB elastase, pyocyanin, rhamnolipid, functional and exotoxin A caused by *Pseudomonas aeruginosa* through changing gene and protein expression [66]. Therefore, SB not only decreased the reproduction of bacteria but also stimulated TH1 induced immune response to accelerate bacterial clearance. Baicalin aluminum complexes can change the whole structure and composition of intestinal microbiome of diarrhea piglets, so as to alleviate diarrhea symptoms. However, its further antibacterial mechanism and regulation of microorganisms have not been clearly described [67]. At present, baicalein aluminum capsule has been used to treat diarrhea.

### Antiviral

T cell infiltration and cytotoxic T-cell-mediated tissue damage have been identified as key factors in RSV disease. Baicalin can reduce T lymphocyte infiltration and pro-inflammatory factor gene expression to play the effect of antiviral [68]. IAV can cause acute lung injury, the specific manifestations were lung index and abnormal lung tissue lesions. SBE significantly improved these lesions [69] by increasing the activity of HA and NA. And the level of inflammatory factors in lung tissue were regulated to inhibit the inflammatory response. SBE can inhibit the propagation of dengue virus, and baicalein is the main active ingredient in the extract [70]. Oroxylin A significantly protects Vero cells from CVB3-induced cell death in vitro, and can improve the symptoms which reduced body weight and blood glucose levels in vivo [71]. 7 μM baicalin can produce directly virus-killing activity against CHIKV in vitro [72], the levels of important protein markers for autophagy and apoptosis were reduced.

### Antioxidant

Wogonin 7-O-β-D-ethylglucuronide, wogonoside and baicalein 7-O-β-D-ethylglucuronide have antioxidant activity [73]. They can inhibit FeSO_4_-Cys-induced lipid peroxidation of liver homogenate, and showed strong cytoprotective effect on H_2_O_2_-induced oxidative damage of human umbilical vein endothelial cells. SBE was found to effectively attenuate comet tail formation and inhibit histone γH2AX phosphorylation induced by H_2_O_2_ [74]. It can also restore the loss of mitochondrial membrane potential by H_2_O_2_ and has the activity of scavenging ROS production in cells. More importantly, SBE blocks the oxidative stress by activating Nrf2/HO-1 signaling pathway to inhibit DNA and cell damage and apoptosis induced by H_2_O_2_. The antioxidant activities of the mixture of polyphenols from four tissues of SB are remarkable [39]. Root has the highest antioxidant activity, followed by leaf, stem and flower. A baicalin/Pluronic F127 hydrogel has excellent cell compatibility and resistance to oxidative stress caused by reactive oxygen species, and accelerate wound healing [75]. In summary, SB has a strong antioxidant effect, and can indirectly exert a cell protective effect through the antioxidant activity.

### Cardiovascular effect

Cerebral ischemia will have neuropathological abnormal symptoms such as neuron loss or swelling, Na^+^-K^+^-ATPase, Ca^2+^-ATPase and the activity of SOD are significantly reduced, and the level of MDA is increased. The flavonoids in SB (35–140 mg/kg) have a significant improvement on the above abnormal conditions, indicating that the flavonoids in SB have a significant therapeutic effect on cerebral ischemia–reperfusion [76]. LysoPC is a membrane phospholipid metabolite that accumulates in ischemic myocardium and plays an important role in the occurrence of ventricular arrhythmias in myocardial dysfunction. Baicalein can protects H9c2 embryonic cardiomyocytes from hemolysin-induced cytotoxicity [77]. It prevented lysoPC-induced cardiomyocyte death, ROS production and the rise of Ca^2+^ concentration in H9c2 cardiomyocyte through the MAPK pathway. In addition, the ratio of Bcl-2/Bax was increased and the expression of cytochrome c, caspase-3, caspase-9 were decreased.

Baicalin can play an anti-hypertensive effect by improving the state of intestinal injury [78]. Intestinal barrier damage plays an important role in the pathogenesis of hypertension. Baicalin can reduce the proximal colonic lesions, intestinal permeability and release levels of related inflammatory factors to achieve anti-hypertensive effect. In addition, SCFA-producing bacteria were induced to increase. Baicalin, baicalein and wogonoside in Sanhuang decoction have vasodilating effects in vitro and anti-hypertensive effect in vivo. It is speculated that these components play roles by activating the NO/cGMP pathway, and the BKCa channel and DAG/PKC/CPI-17 pathway are also involved [79]. Furthermore, baicalin relaxed blood vessels via regulating intracellular Ca^2+^ in vascular smooth muscle and activating ATP-sensitive potassium channel channels [80].

### Anti-diabetics

SB has great therapeutic potential for T2DM, and its flavonoids are the main components that play a role [81]. α-glucosidase inhibitors are currently widely used in the treatment of T2DM. SB contains the above effective ingredients, including 5,7,3,2′,6′-pentahydroxyflavanone, baicalin, viscidulin III, 2′,3,5,6′,7-pentahydroxyflavanone, etc. [82, 83]. They mainly affected the peroxisome proliferator activated receptor signaling pathway in the development of T2DM. PPARγ, PGH2, ACACβ and NF-κB subunit 1 are key targets. Other studies had shown that SBE can play an anti diabetes effect by regulating the composition and structure of intestinal microbial. After oral administration of SBE, the composition of intestinal flora, fecal metabolites and SCFAs content changed in T2DM rats [84].

### Neuroprotective effect

Wogonin has a neuroprotective effect on the rat brain after γ-irradiation [85]. It could restore the mRNA level of cells and the expression of Nrf2, HO-1 and NF-κB. And the lesions of brain tissue structure, such as focal glial degeneration and aggregated astrocytes, also could be treated. SBE can effectively reduce the spinal cord neurons/glial cells and microglial cells peroxide damage and LPS stimulation of injured spinal cord neurons [86]. In LPS-stimulated BV-2 mouse microglia, apigetrin can significantly decrease the production of TNF-α, IL-6, PGE2 and NO and inhibit the expression of COX-2, iNOS and mRNA [87]. Apgetrin, wogonin and baicalein could inhibit neuronal cell death [88, 89]. SBE could inhibit excitotoxicity induced by lactate dehydrogenase and glutamate, and it also had a stronger inhibitory effect and selectivity on NMDA receptor-mediated toxicity [90]. It suggested that SBE had NMDA receptor-mediated neuroprotective effect in excitotoxic neurons. Flavonoids in stem and leaf of SB could dramatically increase cell survival rate, the activities of SOD, glutathione peroxidase, and Na^+^-K^+^-ATPase, inhibit cell apoptosis and excessive production of MDA in primary cortical neurons exposed to potassium cyanide [91]. In summary, the polyhydroxy structure of flavonoids can protect the brain from hypoxia caused by potassium cyanide or cerebral ischemia and inhibit neuronal apoptosis possibly through neuroinflammation, oxidative stress and nerve injury mutual regulation. And it may have potential preventive effects on neurodegenerative diseases.

### Anticancer

Baicalein showed anti-bladder tumor activity [92]. It could reduce the expression of cyclin B1 and D1 by inhibiting protein synthesis and activation via proteasome degradation and inhibit the expression and activity of MMP-2 and MMP-9 mRNA. SBE can inhibit the proliferation of MCF-7 by inhibiting mitochondrial membrane potential, down-regulating Bcl-2/Bax [93]. SBE could downregulate the expression of caspase family members (e.g. PARP), inhibit proliferation and reduce the mitochondrial membrane potential of AGS cells to induce apoptosis [94]. And it would not show obvious toxicity to normal cells. Cisplatin is an important drug for the treatment of lung cancer, but there are serious side effects (such as severe cachexia and acute kidney injury). In combination with SBE, anticancer effect was enhanced and improved the side effects caused by cisplatin therapy in vivo [95]. Baicalin which is the main component of the SBE possess antitumor activity against all leukemic cell lines especially those with MLL and PBX1 gene rearrangements. Baicalin inhibited cell proliferation, arrested the cell cycle at the G0/G1 phase, and induced cell death through caspase 3/7 activation [96]. Oroxylin A could down-regulate the expression of SHCBP1 (it is an oncogene involved in the development of various cancers) to inhibit the carcinogen-induced malignant transformation of JB6 P + skin epidermal cells and decrease the number of tumor cells [97].

### Hepatoprotective effect

Liver cancer is one of the common cancers, and studies had shown that SB and its active ingredients have great potential effect in the treatment of liver cancer [98]. Baicalein could inhibit the proliferation of HepG2 cells and reduce metastasis by regulating the activity of MMP-2 [99]. In addition, it also could down-regulate the expression of mRNA and protein of CD24 (a key protein in cancer cell proliferation) to induce apoptosis of liver cancer cells [100]. SBE could down-regulate the expression of endoplasmic reticulum stress marker GRP78, and had obvious hepatoprotective effect on acute alcohol-induced liver injury [101]. It could regulate the levels of AST, ALT and TG in the serum, and the levels of GSH and MDA in liver tissues. For non-alcoholic fatty liver, Baicalin down-regulated the NLRP3–GSDMD pathway to inhibit liver cell death, but induced cytotoxicity with a doses of 32 μM [102].

### Immunization effect

SBE increased the viability of far eastern catfish (*Silurus asotus*, infected with *Vibrio anguillarum* or *Streptococcus iniae*) by regulating growth and serum hormone levels [103]. SBWE restored the level of Th2-type IgG1 and Th1-type IgG2a of ligation-induced periodontitis mice through immune response [104]. *Haemophilus parasuis* can cause a chronic disease related to inflammatory immune response. Baicalin could inhibit the production of IL-6, IL-8, IL-10 and TNF-α and the phosphorylation of ERK, JNK and p38. Thereby activating the immune response induced by Th-1 to promoting the elimination of bacteria [105]. To sum up, SB play roles of anti-inflammatory, antibacterial and anti-viral via immune response.

#### Other pharmacological effects

##### Anti-aging

SBE improved aging symptoms induced by D-galactose, including the learning and memory function, the oxidative damage and histological abnormalities of the hippocampal neurons [106]. It could regulate the disorder of the metabolism of amino acid, glucose and choline. SB flower extract improved the spatial learning and memory ability, regulated the levels of d-glutamine, glutamic acid, MDA, SOD and AGEs [107]. Therefore, SB flower exerted anti-aging effect through regulating glutamate-glutamic acid metabolism pathway. In addition, 5,7,2′-Trihydroxyflavone and scutevulin inhibited senescence-associated secretory phenotype caused by bleomycin with decreasing the expression of IκBζ and C/EBPβ protein [108]. They didn’t affect either BrdU uptake or the expression of senescence markers.

##### Anti-Osteoporosis

Tectochrysin could significantly improve the loss of bone trabeculae, reduce bone in serum and decrease CTX-1, TRACP-5b and IL-6 levels to relieve the symptoms of Osteoporosis [109]. SB ether extract (Baicalein and wogonin) could promote osteogenic transformation, further bone regeneration, stromal calcification and calcified nodule formation [110]. And their activity is comparable to SIM (0.1 mg/L). Baicalin could affect bone metabolism by promoting osteoblast differentiation, inhibiting osteoclast formation, and increasing osteoclast apoptosis [111, 112].

##### Anti-prostatic hyperplasia

SBE improved significantly prostate growth, and increase serum testosterone and 5α-reductase levels in prostatic hyperplasia rats by inhibiting the expression of AR and proliferating cell nuclear antigen, restoring the balance of Bcl-2/Bax [113]. These findings enhanced the feasibility of plant extracts to replace commercial 5α-reductase inhibitors (such as finasteride).

##### Anti-Alzheimer

MAO-A and MAO-B are considered to treat depression and anxiety drug targets for neuropsychiatric diseases such as Alzheimer and Parkinson [114]. Wogonin and baicalein were observed as effective and selective MAO-A inhibitor [115]. They might be useful lead compound for the development of MAO inhibitors for the treatment of depression, such as Parkinson and Alzheimer. Furthermore, baicalin could promote the differentiation of neurons, which transformation into mature neurons and their survival via the Akt/FOXG1 pathway to exert antidepressant effects [116].

##### Anti-melanin

O-methylated flavones (baicalin, wogonoside, baicalein, wogonin, and oroxylin A) have a dual-function effect on melanocytes, which are the inhibition of melanin production and intracellular melanosome transport [117]. These flavonoids had structure-specific a-ring and aglycon O-methyl. It indicated that the function of the active ingredient is related to its structure, and the structure–activity relationship is reflected.

##### Anti-pruritic

Baicalin, baicalein and oroxylin A could improved histamine-induced scratching behavior through reducing vascular permeability and contraction [118].

## Pharmacokinetics

Decoction is the main form of TCM used in clinic. Baicalein and wogonin were the major active components in Huangqin decoction [119]. After entering the body, their would be metabolized into glucuronidase and sulfatase forms. After oral administration, the AUC_0-t_ of glucuronides/sulfates of wogonin and baicalein reached peak at 10 min. And in serum the content of baicalein and wogonin were ranged from 0.3 to 20 and 0.2 to 10 μg/mL, the C_max_ and AUC_0-t_ of baicalein’s glucuronides/sulfates at each time point were 3.3 times that of wogonin. The second peak of AUC_0-t_ appeared, indicating the occurrence of enterohepatic circulation. The tissues distribution of free baicalein and wogonin were mainly lung and liver respectively. Glucuronides/sulfates of baicalein and wogonin mainly existed in live and kidney respectively. While baicalein and wogonin were mainly present in tissue, their glucuronides/sulfates mainly existed in serum. It suggested that glucuronides/sulfates were involved in circulation. The above components were not detected in brain, suggesting that the above-mentioned components taken orally cannot enter the central nervous system.

SBWE contains mainly 8 flavonoids, baicalin, wogonoside, oroxyloside, norwogonoside, baicalein, wogonin, oroxylin A, norwogonin. After incubation with intestinal bacteria, the content of the latter four components increased significantly, norwogonin had the highest content and showed the highest activity of hemolysis inhibition on sheep and rabbit erythrocytes and anti bacteria while other components didn’t work [120]. Another study showed that under pathological conditions intestinal bacteria had stronger glucuronidase activity and a higher efficiency in converting SBE to flavonoid aglycones [121]. It indicated that SB can exert pharmacological effects depending on the metabolism of intestinal bacteria to produce effective metabolites.

OG and OS were two metabolites of [122] oroxylin A. OA, OG and OS were quickly and widely distributed in tissues, especially postoperative tissues. OA is more widely distributed in tissues than its metabolites, mainly in liver and kidney. But OA was quickly eliminated in the body and the relative bioavailability was less than 2%. The AUC_0-t_ values of them were proportional to dose. After oral administration, OA was mainly excreted from feces, OG was mainly excreted from bile and urine, and OS was almost not excreted.

Clinical data indicated that 100–2800 mg of baicalein in a single oral dose for healthy volunteers is well tolerated, and showed non-toxicity in liver or kidney [123]. And clinical trials had also verified the safety and tolerability it of products currently sold on the market. In summary, most of the ingredients of SB must be metabolized into active compounds by the intestinal flora, such as baicalein and wogonin. In addition, it also shows that some effective ingredients of SB have low oral absorption and availability, short biological half-life, etc. Therefore, how to improve the bioavailability of SB will be the main focus for the follow-up researchers.

## Toxicity

At present, it is generally believed that Chinese herbal medicines have serious side effects, including interstitial pneumonia and liver dysfunction [124]. High dose of wogonin (40 mg/kg, intravenous injection) significantly increased weight of pregnant mice and structural chromosomal aberrations to affect fetus development [125]. Baicalin inhibited the proliferation of targeted stem cells D3 and 3T3 cells, to exert weak decomposition toxicity [126]. Shuanghuanglian injection had a sensitizing effect, and the allergen component was baicalin [127]. It could activate mast cells and increase the levels of IgE and IgG antibody to cause allergic reaction [128]. It indicated that baicalin could produce specific antibodies IgG and IgE in serum, and thus producing allergic reaction. Studies have also shown that baicalin can induce IgE-mediated pseudo-allergy via Mrgprb2 [129]. Baicalin activated TGF-β/Smad signaling pathway to increase kidney collagen synthesis and fibrosis-related protein expression to cause kidney damage and renal fibrosis [130].

**Table 2:** The pharmacological effects of SB

Pharmacological effects	Model	Mechanism	Drugs or components	Doses	Efficacy	Refs.
Anti inflammatory	LPS-induced Raw 264.7 cells	Inhibiting the release of inflammatory factors	Flavonoids	10–100 μg/mL	MC = 10 μg/mL	[59]
	HCT 116 cells	Inducing apoptosis; Activating PPARγ to inhibit the activity of NF-κB	Baicalein	25–100 μM	IC_50_ = 50 μM	[60]
	AOM/DSS-induced colon cancer mices			1–10 mg/kg	MC = 1 mg/kg	
	L6 cells	Inhibiting the expression of inflammation related proteins	Flavonoids	30––150 μg/mL	MC = 150 μg/mL	[61]
	AET II cells	Inhibiting the signal pathways of NF-κB, MAPK and PI3K-AT	70% ethanol extract	3.125–200 μg/mL	MC = 50 μg/mL	[62]
	ALI rats			2–8 mg/kg	MC = 4 mg/kg	
	Human THP-1 cells	Inhibiting the production of TNF-α, IL-1β	Ploysaccharides	20–1280 μg/mL	IC_50_ = 40 μg/mL	[51]
	C57BL/6 mices			50–200 mg/kg	IC_50_ = 100 mg/kg	
Antibacterial and antimicrobial	*T. gondii* in Hela cells	Inhibiting the propagation of *T. gondii*	Water extract	10 μg/mL	Inhibiting rates > 98%	[63]
	Piglets with diarrhea	Changing the composition of intestinal flora	Baicalin-Alumium complexes	272 mg/mL	Diarrhea rates less than 50%	[108]
	Multidrug-resistant *A. baumannii*	Inhibiting the propagation of *A. baumannii*	Water extract	7.8125–1000 μg/mL	MIC = 128 μg/mL MBC = 256 μg/mL	[64]
	*P. Acnes* in human monocytic Th-1 cells	Inhibiting the production of IL-18, IL-6, TNF-α and IL-1β	Wogonin	5–30 μM	IC_50_ = 4.9–8.7 μM	[65]
Antibacterial and antimicrobial	*P. acnes*	Inhibiting the production of IL-8 and IL-1β via the MAPK and NF-κB signaling pathways	Wogonin Wogonoside	1.15 mg/g 8.71 mg/g	Inhibition rates > 90%	[66]
	*P. aeruginosa*	Decreasing the production of exotoxin A; Inhibiting inflammation	Baicalin	2–1024 μg/mL	MEC > 1024 μg/mL	[67]
	*P. aeruginosa*-induced peritoneal infection mouse			100 mg/kg	Bacterial counts decreased significantly	
Antivirus	RSV infect HEp-2 cells	Decreasing inflammatory cell infiltration; Significantly reducing H1N1 activity	Baicalin	3–30 μM	IC_50_ = 19.9 ± 1.8 μM CC_50_ = 370 ± 10 μM	[68]
	RSV-induced lung injury mice			50–200 mg/kg	IC_50_ = 100 mg/kg	
	IAV in MDCK cells	Inhibiting the leves of IL-6, TNF-α; Improving lung tissue abnormality	Flavonoids	2.5–40 μg/mL	Inhibiting rates > 65%	[69]
	IAV in mices			10 mg/mL		
	Dengue virus in vero cells	Inhibiting the propagation of virus	Baicalein	0.5–750 μg/mL	IC_50_ = 56.02–77.41 μg/mL	[70]
	CVB3 in male BALB/c mices	Reducing the level of inflammatory factors; Increasing phosphoric acid eIF2 α in pancreas	Oroxylin A and wogonoside	50 mg/kg	Inhibiting rates > 50%	[71]
Antivirus	CHIKV in vero, BHK-21 and HEK-293T cells	Antivirus directly; Reducing the level of important protein markers of LC3 and Bax	Baicalin	3–100 μM	EC_50_ = 14 μM	[72]
Antioxidant	FeSO_4_-Cys-induced liver homogenate	Inhibiting further development of oxidation process; Producing cytoprotection	wogonin-7-O-β-d-ethylglucuronide and wogonoside	–	IC_50_ = 18.2 and 24.9 μM	[73]
	H_2_O_2_-induced human umbilical vein endothelial cells					
	H_2_O_2_-induced HaCaT cells	Decreasing comet tail formation; Inhibiting histone γH2AX phosphorylation	Ethanol extract	200–1000 μg/mL	IC_50_ = 600 μg/mL	[74]
	DPPH^−^, ABTS^2+^ scavenging activity	Inhibiting oxidation reaction	Polyphends	25–500 μg/mL	IC_50_ = 66.9 ± 0.3 μg/mL	[39]
	DPPH^−^ scavenging activity	Inhibiting oxidation reaction; Preventing ROS from damaging cells; Accelerating wound healing	Baicalin/F 127 hydrogels	–	Wound healing rates more than 85% and cell activity more than 80%	[75]
	NIH3T3 cells					
	Wound tissues					
Cardiovascular effects	Cerebral ischemial reperfusion rats	Improving neuron loss or swelling; Enhancing memory; Reducing MDA level; Increasing Na^+^–K^+^–ATPase activity	Flavonoids	35–140 mg/kg	MC = 35 mg/kg	[76]
	H9c2 cardiomyoctes	Reducing LysoPC-induced cell desth and production of ROS	Baicalein	0.1–10 μM	IC_50_ = 0.69 μM	[77]
Cardiovascular effects	Age-matched male SHRs and Wistar-Kyoto rats	Improving the state of intestinal injury, Reducing intestinal permeability and the level of related inflammatory factors	Baicalin	100 mg/kg	The level of correlation factor is 2–5 times higher than that of model making	[78]
	Male SD rats, male WKY/Izm and SHRs rats	Activating the pathway of NO/cG; Paying the role of vascular relaxation	Sanhuangjiedu Decoction	1–100 μg/mL	EC_50_ = 16.2 ± 1.1 and 65.1 ± 5.5 μg/mL (baicalin and baicalein)	[79]
Hypoglycemic	α-Glucosidase	Inhibiting the activity of α-glucosidase	70% Ethanol extract	–	Six flavonoids of α-glucosidase inhibitors were screened out	[83]
	Male SD rats	Regulating the composition of intestinal flora	Water extract	6.3 g/kg	The perturbation of metabolic spectrum in T2DM rats was significantly improved	[84]
	α-Glucosidase	Regulating peroxisome proliferator receoter actiated	Flavonoids	3.34 mg/kg	Inhibiting rates > 90%	[85]
Neuroprotection	γ irradiation-induced rats	Resuming the level of cell mRNA and the expression of Nrf2, HO-1 and NF-κB	Wogonin	30 mg/kg	No lesions of the polymerized astrocytes	[85]
	Spinal cord injury rats	Reducing peroxides toxicity in nerve cells	Water extract	20 mg/kg	The pathological changes of damaged neurons were improved obviously	[86]
Neuroprotection	LPS -induced BV-2 and HT22 cells	Inhibiting neuroinflammatory responses	Apigetrin	20–100 μM	MC = 25 μM	[87]
	Hippocampal neuroral cells	Decreasing the production of PEG2 and NO	Wogonin, baicalein, wogonoside and baicalin	10 mg/kg	Inhibiting rates are 78.6%, 91.0%, 81.0% and 41.0%	[88, 89]
	Primary rats cortical cells	Inhibition of neurocell death	Ethanol extract	1–100 μg/mL	IC_50_ = 35.1 μg/mL	[90]
	Primary rats cortical cells exposured to potassium cyanide	NMDA receptor mediated neuroprotection; Inhibiting lactic dehydrogenase, MDA etc.; Increasing the Na+–K+–ATPase activity	Flavonoids	18.98–75.92 μg/mL	Inhibiting rates are 25.24–46.69%	[91]
Antitumor	Mouse orthotopic tumor model and femal C57BL/6 mices	Decreasing the expression of cyclin B1 and D1; Inhibit the mRNA expression of MMP-2 and MMP-9	Baicalein	25–100 μM	MC = 100 μM (G1 phase)	[92]
	MCF-7	Inhibition of mitochondrial membrane potential; Downregulating Bcl-2	Methanol extract	100–500 μg/mL	MC = 100 μg/mL	[93]
	B-ALL cell lines	Inhibiting cell proliferation, arrested the cell cycle at the G0/G1 phase	Baicalin	4–16 μg/mL	MC = 8 μg/mL	[96]
	AGS cells	Inhibition of mitochondrial membrane potential; Downregulating PARP	Flavonoids	50–400 μg/mL	IC_50_ = 100 μg/mL	[94]
Antitumor	Lewis lung cacinoma cells	Inhibiting the propagation of AGS cells and tumor growth	Extract freeze-dried powder	0.125–1 mg/mL	IC_50_ = 0.13 mg/mL	[95]
	Male C57BL/6 mices			300 mg/kg		
	Female ICR mices	Inhibiting the expression of SHCBP1	Oroxylin A	10–40 mg/kg	MC = 10 mg/kg	[97]
	JB6P + cells			5–20 μM	MC = 5 μM	
Liver protection	HCC cells	Inhibiting the metastasis of cancer cells	Flavonoids	50–400 μg/mL	MC = 100 μg/mL	[99]
	HCC cells	Downregulating the mRNA and proteins expression of CD24	Baicalein	50–100 μg/mL	MC = 50 μg/mL	[100]
	Alcohol-induced acute liver injury in mices	Inhibiting the propagation of liver cancer cells; Downregulating the GRP78 expression of endoplasmic reticulum marker	Methanol extract	40–160 mg/kg	MC = 40 mg/kg	[101]
	Non-alcoholic steatohepatitis cells	Downregulating the pathway of NLRP3-GSDMD to inhibit the liver cells death	Baicalin	1–64 μM	MC = 32 μM	[102]
Immunity	Far eastern catfish	Increasing viability by regulating growth and serum hormone levels	Water extract	0.25–5%	Viability more than 90%	[60]
	Srtreptococcus iniae induced periodontitis mices	Increasing the level of Th2-type IgG1	Water extract	50 mg/kg	After 4 weeks, the effect began to be obvious	[104]
Immunity	PAVECs	Anti *H. parasuis*; Inhibiting the phosphorylation of FPK, JNK, p38	Baicalin	12.5–100 μg/mL	MC = 12.5 μg/mL	[105]
Anti-aging	d-galactose induced aging rats	Regulatiing disorders of amino acid, choline and glucose metabolism	60% Ethanol extract	100–200 mg/kg	MC = 100 mg/kg	[106]
	d-galactose induced aging rats	Regulating the level of MDA, SOD, AGEs	60% Ethanol flower extract	0.4–0.8 g/kg	MC = 0.4 g/kg	[107]
	Bleomycin-induced senescence in BJ fibroblasts	Interrupting IκBζ/C/EBPβ pathway	5,7,2′-Trihydroxyflavone and scutevulin	2-4 mg/kg	Inhibitory rates > 90%	[108]
Anti-Osteoporosis	Primary bone narrow mononuclear cells	Inhibition of trabecular bone loss; Decreasing the level of CTX-1, TRAP-5b and IL-6	Tectochrysin	20–100 μmol/L	MC = 20 μmol/L	[109]
	Female C57BL/6 mices	Promoting the proliferation of bone cells and matrix calcification	Baicalein and wogonin	0.5–0.6 mg/L and 0.015–0.6 mg/L	The effect is equivalent to 0.1 mg/L simvastatin	[110]
Anti prostatic hyperplasia	Prostatic hyperplasia rats	Inhibiting prostate growth; Decreasing the level of serum testosterone and 5α-reductase	30% ethanol extract	100–200 mg/kg	MC = 100 mg/kg	[113]
Anti-alzheimer	MAO enzyme	Inhibiting MAO-A and MAO-B	Wogonin	–	IC_50_ = 6.35 and 20.8 μM (A and B)	[115]
Anti-melanin	The mouse melanoma cell line B16F10	Inhibiting the production of melanin and transport of intracellular melanosome	O-methylated flavones	7–70 μg/mL	MC = 35 μg/mL	[117]
Antipruritic	Male ICR and BALB/c mices	Inhibiting scratching behavior; Reducing vascular permeability	Baicalin, baicalein and oroxylin A	20–50 mg/kg	Oroxylin A has the strongest effect	[118]

SB has more applications in modern skin care industry due to its antioxidant, anti-inflammatory and melanin synthesis inhibitory effects. But the introduction of botanical preparations into cosmetics is an increasing cause of contact dermatitis in patients. There were studies reported that sunscreen containing SB could cause facial inflammation to somebody [131–133]. Therefore, whether SB can be widely used in cosmetics or skin care products still needs further study.

## Probably potential therapeutic effect and mechanism of COVID-19

COVID-19 is a worldwide and severe epidemic at present, caused by SARS-CoV-2 [134]. A research team pointed out that the SARS-CoV-2 virus is similar to the SARS coronavirus [135]. It is suggested that the therapeutic target of SARS can be used as a reference for treatment strategy. Researched showed that angiotensin converting enzyme 2 (ACE2) and coronavirus 3CL Mpro on host epithelial cells affected by its S-protein are considered to be the core targets for inhibiting coronavirus proliferation [136, 137]. Simultaneously, cytokine storm induced by virus is the main cause of complications, such as inflammation, septic shock and multiple organ failure [138].

**Fig. 3:**
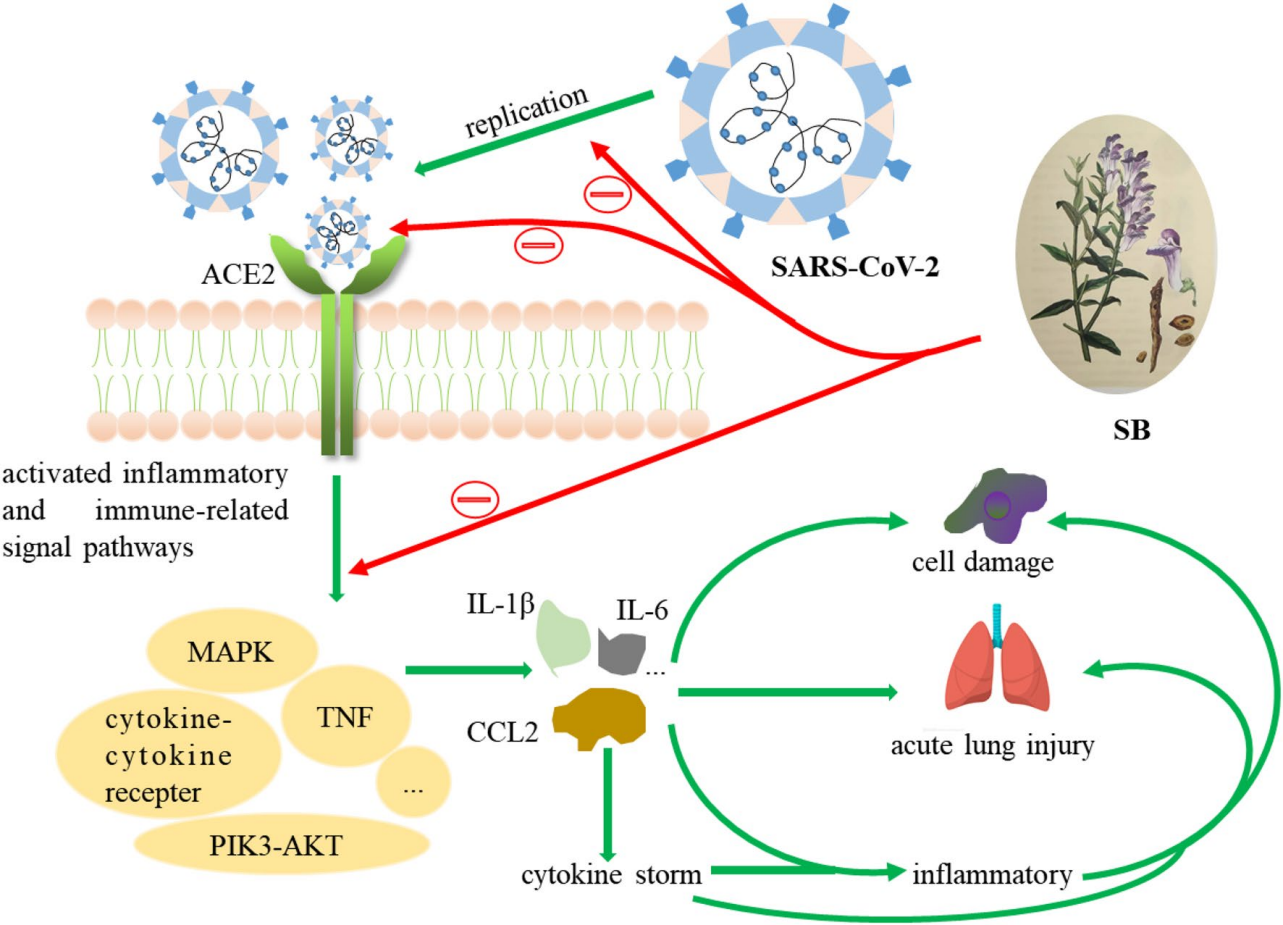
Mechanisms of SB in treating COVID-19

Baicalin had been confirmed to inhibit SARS-CoV in vitro [139], and scutellarin could interact with ACE2 [140]. At present, the drug research on the treatment for COVID-19 is mainly based on network pharmacology and molecular docking [141]. Baicalein and oroxylin A have a certain binding activity with ACE2 and 2019-nCoV-M^pro^, indicating that they may directly act on the virus and host cells, thus preventing virus proliferation, preventing the body’s immunity and blocking virus attack [142–144]. Naringenin and beta-sitosterol can regulate the expression of key genes (CCL2, IL-1β and IL-6) in the treatment of COVID-19, and produce anti-inflammatory and immune enhancing effects through IL-17, TNF, AGE-RAGE signaling pathways and cytokine-cytokine recepter interaction pathway [141]. It is speculated that the therapeutic effects of compounds of SB on COVID-19 mainly focus on anti-inflammatory, inhibiting pro-inflammatory cytokine production and cut of cytokine storm, regulating immune response. Mechanisms of SB in treating COVID-19 shown in Fig. 3. At present, the treatment for COVID-19 researches mainly focus on TCM prescriptions. In addition to the above mentioned, Lianhua Qingwen can regulate the imbalance of ACE-Ang-II and ACE2-Ang-(1-7), which can lead to overwhelming pro-inflammatory cytokines with cytokine storm. And regulating immune-related signal pathway (MAPK, NF-κB, PI3K-AKT, ect) to protect organ damage [145].

To sum up, TCM exhibit functions on COVID-19 via “multi-component, multi-target and multi-pathway”. Some countries authorized chloroquine and hydroxychloroquine for the treatment of COVID-19 [146]. But they have adverse reactions, such as diarrhea and nausea, so it is particularly important to seek treatment from TCM. In China, there are three formula authorized to treat COVID-19 [147, 148], including Jinhua Qinggan granules, Lianhua Qingwen granules, and Xuebijing injection. At present, the development of accurate and effective therapeutic drugs and vaccines for COVID-19 are the research focus of various countries [149]. Although TCM has many advantages mentioned above, its exact effects still needs to be verified by clinical trials.

## Conclusion and future perspectives

SB is a common TCM with a wide range of clinical effects, and usually used to treat cold, cough, dysentery, lung heat, jaundice and other diseases. According to botanical research, SB in ChP. (1st, 2020) is authentic. In addition to genuine factors, harvest time and processing technology also affect the efficacy of herbs [150]. Despite the commercial interest and increasing demand for SB, improvements through breeding have been very limited. The absence of genome information has limited the understanding of how its flavonoid bioactivities are made and have limited any improvement in productivity through genetic selection. Understanding the genes responsible for biosynthesis of the various flavonoids made in S. baicalensis and their regulation will lay a foundation for molecular breeding for improved, sustainable production.

SB contains a variety of flavonoids, which are the material basis for its strong biological activity, such as baicalin, baicalein, oroxylin A, wogonin, norwogonin and so on. But beyond that, SB also has diterpenes, polyphenols, amino acids, volatile oils, sterols, benzoic acids, etc. Therefore, it have many pharmacological functions such as antibacterial, antiviral, anti-inflammatory, anticancer, liver-protecting and neuroprotective effects. However, the effective components of SB showed low bioavailability and rapid metabolism in vivo. It’s suggested that for SB oral preparation, it is necessary to study more suitable technologies to improve its bioavailability in vivo in order to achieve better curative effect. Baicalin is the allergen in SB, which can cause the allergic reaction mediated by IgG and IgE. Therefore, the injection and cream containing baicalin should be strictly controlled to ensure its safety. SB also has good application prospects in non-medical fields, such as agriculture, industry and beauty industry. Because SB can inhibit the production and transportation of melanin, it is added more in whitening skin care products. The introduction of plant preparations into cosmetics is a relatively risky measure, which may cause adverse reactions to users, so its production needs to be strictly controlled.

Baicalin and baicalein had been proved to have inhibitory effect on SARS-CoV in vitro, and scutellarin could also bind with ACE2 receptor to prevent virus invasion. At the same time, they alleviated the complications caused by the virus, through anti-inflammatory, improve immune response and other functions. It is worth noting that the virus will affect the body through multiple pathways and cause many complications. Therefore, compared with the single component, the TCM prescriptions are still the main treatment for COVID-19, and the therapeutic characteristics of “multi-component, multi-target and multi-pathway” of TCM are brought into full play. Due to the lack of understanding of the pathogenesis of COVID-19, symptomatic treatment and alleviation of complications are the main treatment strategies before the development of effective drugs and vaccines. In conclusion, the current researches of SB are summarized, so that readers have a comprehensive understanding of the research extent of SB, and provide ideas for the follow-up study of SB, especially in how to improve the bioavailability of SB oral preparations in vivo.

## Data Availability

Not applicable.

## Abbreviations

PAL: Phenylalanine ammonia lyase
C4H: Cinnamate 4-hydroxylase
CoA: Coenzyme A
CLL: 4-Coumarate CoA ligase
CHS: Chalcone synthase
CHI: Chalcone isomerase
FNSII: Flavone synthase II
MT: Methyltransferases
GT: Glycosyltransferases
FH: Flavone hydroxylase
OMT: O-methyltransferases
DSS: Dextran sodium sulfate
LPS: Lipopolysaccharide
iNOS: Inducible nitric oxide synthase
COX: Cyclooxygenase
IL: Interleukin
TNF-α: Tumor necrosis factor-alpha
AOM: Azoxymethane
PPARγ: Peroxisome proliferator-activated receptor gamma
NF-κB: Nuclear factor-kappa B
AET II: Alveolar epithelial type II
SBE: SB ethanol extracts
SBWE: SB water extract
MAPK: Mitogen activated protein kinase
MIC: Minimum inhibitory concentration
MBC: Minimum bactericidal concentration
MC: Minimal concentration
ED_50_: Median effective dose
IC_50_: Half maximal inhibitory concentration
HA: Hemagglutinin
NA: Neuraminidase
H2AFX: H2A histone family member X
ROS: Reactive oxygen species
Nrf2/HO-1: Nuclear factor-erythroid 2-related factor 2/Heme oxygenase 1
SOD: Superoxide dismutase
MDA: Malondialdehyde
LysoPC: Lysophosphatidylcholine
Bcl-2: B-cell lymphoma 2
Bax: Bcl-2-associated X protein
SCFAs: Short-chain fatty acids
NO/cGMP: Nitric oxide/cyclic Guanosine monophosphate
BKCa: Large conductance calcium-activated potassium channels
DAG/PKC/CPI-17: Diacylglycerol/Protein kinase C/Cytosolic protein of 17 kDa
T2DM: Type 2 diabetes
PGH2: Prostaglandin G/H synthase 2
ACACβ: Acetyl-CoA carboxylase beta
PGE2: Prostaglandin E2
NMDA: N-methyl D-aspartate
MMP: Matrix metalloproteinase
PARP: Poly (ADP-ribose) polymerase
FOXM1: Forkhead Box M1 activity
AST: Aspartate transaminase
ALT: Alanine transferase
TG: Triglyceride
GSH: Glutathione
GRP78: 78-kDa glucose-regulated protein
NLRP3-GSDMD: NLR pyrin domain containing 3-gasdermin D
PAVECs: Porcine aortic vascular endothelial cells
JNK: c-JUN N-terminal kinase
ERK: Extracellular signal-regulated kinase
AGEs: Advanced glycation end products
SIM: Simvastatin
CTX-1: TRACP-5b: Bone turnover markers
MAO: Monoamine oxidase
ALI: Acute lung injury
OG: Oroxylin A 7-O-glucuronic acid
OS: Oroxylin A sodium sulfonate
D3: Embryonic stem cell
3T3: Embryonic fibroblast
TGF-β: Transforming growth factor-β
Smad: The main signal transducers for receptors of the TGF-β
SARS-CoV-2: Severe acute respiratory syndrome coronavirus 2
BrdU: 5-Bromo-2′-deoxy-uridine
